# Cerebral venous thrombosis in a young asymptomatic COVID-19 patient

**DOI:** 10.1097/j.pbj.0000000000000165

**Published:** 2022-09-09

**Authors:** Rafael Dias, Ricardo Soares dos Reis, Sara Pereira de Sousa, Ana Filipa Rocha, Ana Margarida Ribeiro, Jorge Almeida

**Affiliations:** a Department of Neurology, Centro Hospitalar Universitário de São João,; b Department of Clinical Neurosciences and Mental Health, Faculty of Medicine of University of Porto,; c Department of Neurology, Hospital Central do Funchal,; d Department of Immunohemotherapy, Centro Hospitalar Universitário de São João,; e Department of Internal Medicine, Centro Hospitalar do Tâmega e Sousa,; f Department of Internal Medicine, Centro Hospitalar Universitário de São João,; g Department of Medicine, Faculty of Medicine of University of Porto

**Keywords:** asymptomatic, cerebral venous thrombosis, COVID-19, SARS-CoV-2

## Abstract

We report an unusual case of cerebral venous thrombosis (CVT temporally associated with an otherwise asymptomatic severe acute respiratory syndrome coronavirus 2 (SARS-CoV-2) infection. While coronavirus disease 2019 (COVID-19) has been associated with thrombotic events at different sites, most published cases report on symptomatic SARS-CoV-2 infection. We are confident this was an otherwise asymptomatic infection as the patient reported no symptoms and molecular and serological testing was consistent with infection more than 3weeks in the past.

We believe this is an important report as it adds to the existing literature on thrombotic events in patients with COVID. It may even inform discussion of COVID vaccines and CVT since our patient, as those reported in association with vaccines, also had thrombocytopenia on admission.

To the Editor,

Coronavirus disease 2019 (COVID-19) is caused by severe acute respiratory syndrome coronavirus 2 (SARS-CoV-2). This infection has been associated with hematological manifestations, including a higher incidence of thrombotic and embolic events occurring in 7.7% to 28.0% of hospitalized COVID-19 patients.^1^ One such manifestation is cerebral venous thrombosis (CVT), a rare subtype of stroke.^1,2^ This condition may be the result of a hypercoagulable state induced by the cytokine storm and/or endothelial damage associated with COVID-19.^1^

We report a 19-year-old female patient who presented to the emergency department with complaints of a progressively worsening left frontal-parietal headache. She had a history of tension-type headaches, smoking, and was taking combined oral contraceptives. The pain had started 4 days prior to presentation and score 8/10 on the numeric pain rating scale. It was not worsened or relieved by postural changes and it was associated with photophobia. The pain was only mildly relieved by non-steroidal anti-inflammatory drugs. The patient denied nausea, vomiting, focal symptoms, or any sign of infection including fever, cough, dyspnoea, thoracic pain, diarrhea, anosmia, or ageusia.

Physical and neurological examinations were normal. Laboratory tests performed showed thrombocytopenia (61.000/μL) de *novo* and elevated C-reactive protein (29.7mg/L). Coagulation tests were normal. Chest X-ray was unremarkable. A plain head computed tomography (CT) was performed and showed a hyperdense left transverse sinus. CT cerebral venography confirmed thrombosis of the left transverse sinus and internal jugular vein. SARS-CoV-2 polymerase chain reaction (PCR) test was negative (Fig. 1).

**Figure 1:**
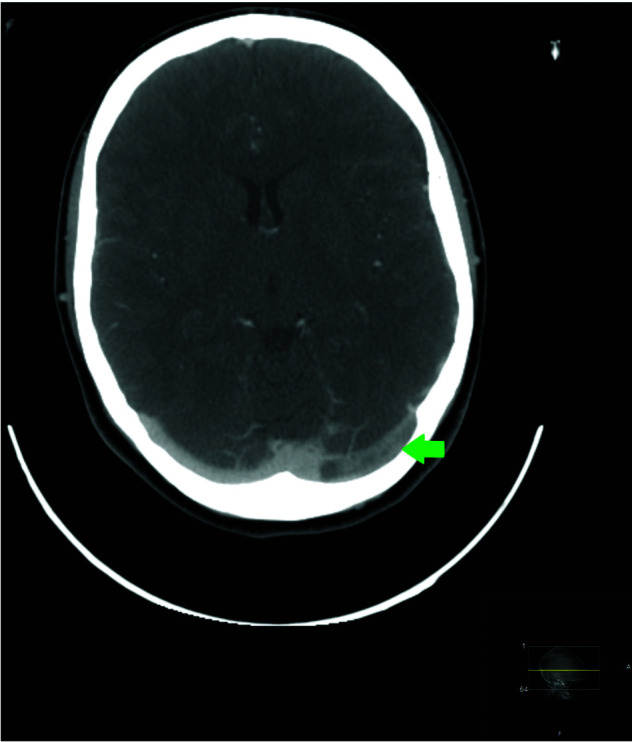
CT cerebral venography demonstrating thrombosis of the left transverse sinus. CT: computed tomography.

The patient was initiated on a therapeutic dose of low-molecular-weight heparin and transferred to the stroke unit of our tertiary center. SARS-CoV-2 PCR repeat testing came back weakly positive at a cycle threshold of 37. Serological testing revealed positive IgG antibodies against SARS-CoV-2 consistent with prior asymptomatic COVID-19 infection. Autoimmune and prothrombotic workup, including anti-PF4 antibodies, was negative.

The patient had an uneventful recovery and platelet numbers normalized. She has discharged on dabigatran 150mg twice daily for 6 months, instructed to stop smoking and to stop taking oral contraceptives.

This case report adds to the current and growing body of literature on CVT as a complication of COVID-19. Surprisingly, in this patient, CVT on an otherwise asymptomatic SARS-CoV-2 infection.

While published cases report that SARS-CoV-2 infection may occur more than 2 weeks before symptoms of CVT begin, consistent with the presentation of our patient, these are mostly reports of moderate or severe COVID-19 infection.^1^ Of note, our patient did not present with any clinical or imaging signs of COVID-19 apart from a weakly positive PCR test and positive

SARS-CoV-2 IgG antibodies. Overall, these findings are consistent with a recent, but resolving asymptomatic SARS-CoV-2 infection.

Even though the patient had other risk factors for CVT, such as smoking and combined oral contraception, COVID-19 may have been the precipitating factor in this case. The patient had concomitant thrombocytopenia, a known complication of COVID-19.^3^ This likely reflects platelet dysfunction and hyper-activation with subsequent thrombus formation.^4^ Indeed, SARS-CoV-2 infection has been associated with a hypercoagulable state and a high incidence of venous thromboembolism.^1^ Similar mechanism have been hypothesized for cases of thrombosis, including CVT, associated with COVID-19 vaccines, although the vaccine-specific risk has yet to be demonstrated.^5^

While CVT is an uncommon cause of stroke, our case report highlights the possible association between totally asymptomatic SARS-CoV-2 infection and the development of CVT in a young female, however further clinical research is warranted. Given that symptoms such as headache may be nonspecific, it is important for clinicians to consider plain CT/CT cerebral venograms in COVID-19 patients presenting with headache and clinical red flags.
